# Supplementary material for: Untargeted Metabolomics Approach Using UHPLC-HRMS to Unravel the Impact of Fermentation on Color and Phenolic Composition of Rosé Wines
Source: Molecules. 2023 Jul 29;28(15):5748. doi: 10.3390/molecules28155748 (PMC10421246; doi:10.3390/molecules28155748)
Supplement: Supplementary file 1 [file molecules-28-05748-s001.zip › File S1.pdf]

File S1: Compound Discoverer WorkFlow.

Processing node 33: Select Spectra

----

1. Spectrum Properties Filter:

- Lower RT Limit: 0.8

- Upper RT Limit: 17

- First Scan: 0

- Last Scan: 0

- Ignore Specified Scans: (not specified)

- Lowest Charge State: 0

- Highest Charge State: 0

- Min. Precursor Mass: 500 Da

- Max. Precursor Mass: 1000 Da

- Total Intensity Threshold: 0

- Minimum Peak Count: 1

2. Scan Event Filters:

- Mass Analyzer: Is FTMS

- MS Order: Any

- Activation Type: (not specified)

- Min. Collision Energy: 0

- Max. Collision Energy: 1000

- Scan Type: Is Full

- Polarity Mode: Any

3. Peak Filters:

- S/N Threshold (FT-only): 1.5

4. Replacements for Unrecognized Properties:

- Unrecognized Charge Replacements: 1
- Unrecognized Mass Analyzer Replacements: ITMS
- Unrecognized MS Order Replacements: MS2
- Unrecognized Activation Type Replacements: CID
- Unrecognized Polarity Replacements: +
- Unrecognized MS Resolution@200 Replacements: 60000
- Unrecognized MSn Resolution@200 Replacements: 30000

5. General Settings:

- Precursor Selection: Use MS(n - 1) Precursor
- Use Isotope Pattern in Precursor Reevaluation: True
- Provide Profile Spectra: Automatic
- Store Chromatograms: False

Processing node 26: Align Retention Times

----

1. General Settings:

- Alignment Model: Adaptive curve
- Alignment Fallback: Use Linear Model
- Maximum Shift [min]: 1
- Shift Reference File: True
- Mass Tolerance: 2 ppm
- Remove Outlier: True

Processing node 9: Detect Compounds

----

1. General Settings:

- Mass Tolerance [ppm]: 2 ppm
- Intensity Tolerance [%]: 50
- S/N Threshold: 3
- Min. Peak Intensity: 500000
- Ions: [2M+H]+1; [M+2H]+2; [M+H]+1; [M-e]+1
- Base Ions: [M+H]+1; [M-H]-1
- Min. Element Counts: C H
- Max. Element Counts: C190 H390 N20 O80

2. Peak Detection:

- Filter Peaks: True
- Max. Peak Width [min]: 0.8
- Remove Singlets: True
- Min. # Scans per Peak: 5
- Min. # Isotopes: 1

3. Isotope Grouping:

- Min. Spectral Distance Score: 0
- Remove Potentially False Positive Isotopes: True

Processing node 31: Group Compounds

----

1. Compound Consolidation:

- Mass Tolerance: 1 ppm
- RT Tolerance [min]: 0.4

2. Fragment Data Selection:

- Preferred Ions: [2M+H]+1; [M+2H]+2; [M+H]+1; [M-e]+1

Processing node 32: Fill Gaps

----

1. General Settings:

- Mass Tolerance: 1 ppm

- S/N Threshold: 2

- Use Real Peak Detection: True

Processing node 42: Apply Missing Value Imputation

----

1. General Settings:

- Imputation Method: Automatic Selection

- Fill Blanks with min value: False

2. Random Forest Settings:

- Number of trees: 100

- Max Number of Iterations: 10

Processing node 43: Apply QC Correction

----

1. General Settings:

- Regression Model: Linear

- Min. QC Coverage [%]: 50

- Max. QC Area RSD [%]: 30

- Max. Corrected QC Area RSD [%]: 25

- Max. # Files Between QC Files: 15

Processing node 44: Mark Background Compounds

----

1. General Settings:

- Max. Sample/Blank: 5

- Max. Blank/Sample: 0

- Hide Background: True

Processing node 29: Predict Compositions

----

1. Prediction Settings:

- Mass Tolerance: 1 ppm

- Min. Element Counts: C H

- Max. Element Counts: C190 H390 N10 O80 S5

- Min. RDBE: 0

- Max. RDBE: 40

- Min. H/C: 0.1

- Max. H/C: 4

- Max. # Candidates: 10

- Max. # Internal Candidates: 200

2. Pattern Matching:

- Intensity Tolerance [%]: 30

- Intensity Threshold [%]: 0.1

- S/N Threshold: 3

- Min. Spectral Fit [%]: 30

- Min. Pattern Cov. [%]: 90

- Use Dynamic Recalibration: True

3. Fragments Matching:

- Use Fragments Matching: True
- Mass Tolerance: 5 ppm
- S/N Threshold: 3

Processing node 25: Assign Compound Annotations

----

1. General Settings:

- Mass Tolerance: 1 ppm

2. Data Sources:

- Data Source #1: Predicted Compositions
- Data Source #2: mzVault Search
- Data Source #3: MassList Search
- Data Source #4: (not specified)
- Data Source #5: (not specified)
- Data Source #6: (not specified)
- Data Source #7: (not specified)

3. Scoring Rules:

- Use mzLogic: True
- Use Spectral Distance: True
- SFit Threshold: 20
- SFit Range: 20

Processing node 17: Differential Analysis

----

1. General Settings:

- Log10 Transform Values: True
